# Author Correction: Bioinformatic and cell-based tools for pooled CRISPR knockout screening in mosquitos

**DOI:** 10.1038/s41467-022-29489-w

**Published:** 2022-03-30

**Authors:** Raghuvir Viswanatha, Enzo Mameli, Jonathan Rodiger, Pierre Merckaert, Fabiana Feitosa-Suntheimer, Tonya M. Colpitts, Stephanie E. Mohr, Yanhui Hu, Norbert Perrimon

**Affiliations:** 1 Department of Genetics, Blavatnik Institute, Harvard Medical School, Boston, MA 02115 USA; 2 Department of Microbiology, National Emerging Infectious Diseases Laboratories, Boston University School of Medicine, 620 Albany Street, Boston, MA 02118 USA; 3 HHMI, Harvard Medical School, Boston, MA 02115 USA

**Keywords:** High-throughput screening, Functional genomics, CRISPR-Cas9 genome editing

Correction to: *Nature Communications* 10.1038/s41467-021-27129-3, published online 24 November 2021.

The original version of this Article contained an error in Fig. 5, in which the *A. coluzzii* ortholog of *PTP-ER* AGAP007118 was incorrectly listed as AGAP028616. In addition, the sentence “Target genes included Anopheles orthologs of FKBP12 (AGAP012184), which encodes the cellular binding partner of the mTOR inhibitor rapamycin; EcR (AGAP028634) and usp (AGAP002095), which encode mediators of an antiproliferative transcriptional response to treatment with ecdysone; and PTP-ER(AGAP028616), which encodes a negative regulator of the mitogen-activated protein kinase (MAPK) signaling cascade that can be suppressed by treatment with the MEK inhibitor trametinib (Fig. 5a; Supplementary Data 3)” was corrected to “Target genes included Anopheles orthologs of FKBP12 (AGAP012184), which encodes the cellular binding partner of the mTOR inhibitor rapamycin; EcR (AGAP028634) and usp (AGAP002095), which encode mediators of an antiproliferative transcriptional response to treatment with ecdysone; and PTP-ER(AGAP007118), which encodes a negative regulator of the mitogen-activated protein kinase (MAPK) signaling cascade that can be suppressed by treatment with the MEK inhibitor trametinib (Fig. 5a; Supplementary Data 3)”.

This has been corrected in both the PDF and HTML versions of the Article.

