# Supplementary material for: Bioinformatic and cell-based tools for pooled CRISPR knockout screening in mosquitos
Source: Nat Commun. 2021 Nov 24;12:6825. doi: 10.1038/s41467-021-27129-3 (PMC8613219; doi:10.1038/s41467-021-27129-3)
Supplement: Supplementary file 1 — Supplementary Information [file 41467_2021_27129_MOESM1_ESM.pdf]

Bioinformatic and cell-based tools for pooled CRISPR knockout screening in mosquitos

Raghuvir Viswanatha[1†*], Enzo Mameli[1,2†], Jonathan Rodiger[1], Pierre Merckaert[1], Fabiana Feitosa-Suntheimer[2], Tonya M. Colpitts[2], Stephanie E. Mohr[1], Yanhui Hu[1], Norbert Perrimon[1,3*]

[1] Department of Genetics, Blavatnik Institute, Harvard Medical School, Boston, MA 02115, USA.

[2] Department of Microbiology, National Emerging Infectious Diseases Laboratories, Boston University School of Medicine, 620 Albany Street, Boston, MA 02118, USA

[3] HHMI, Harvard Medical School, Boston, MA 02115, USA.

[†] Equal contributions

*Correspondence: perrimon@receptor.med.harvard.edu
ram@genetics.med.harvard.edu

SUPPLEMENTARY FIGURE 1

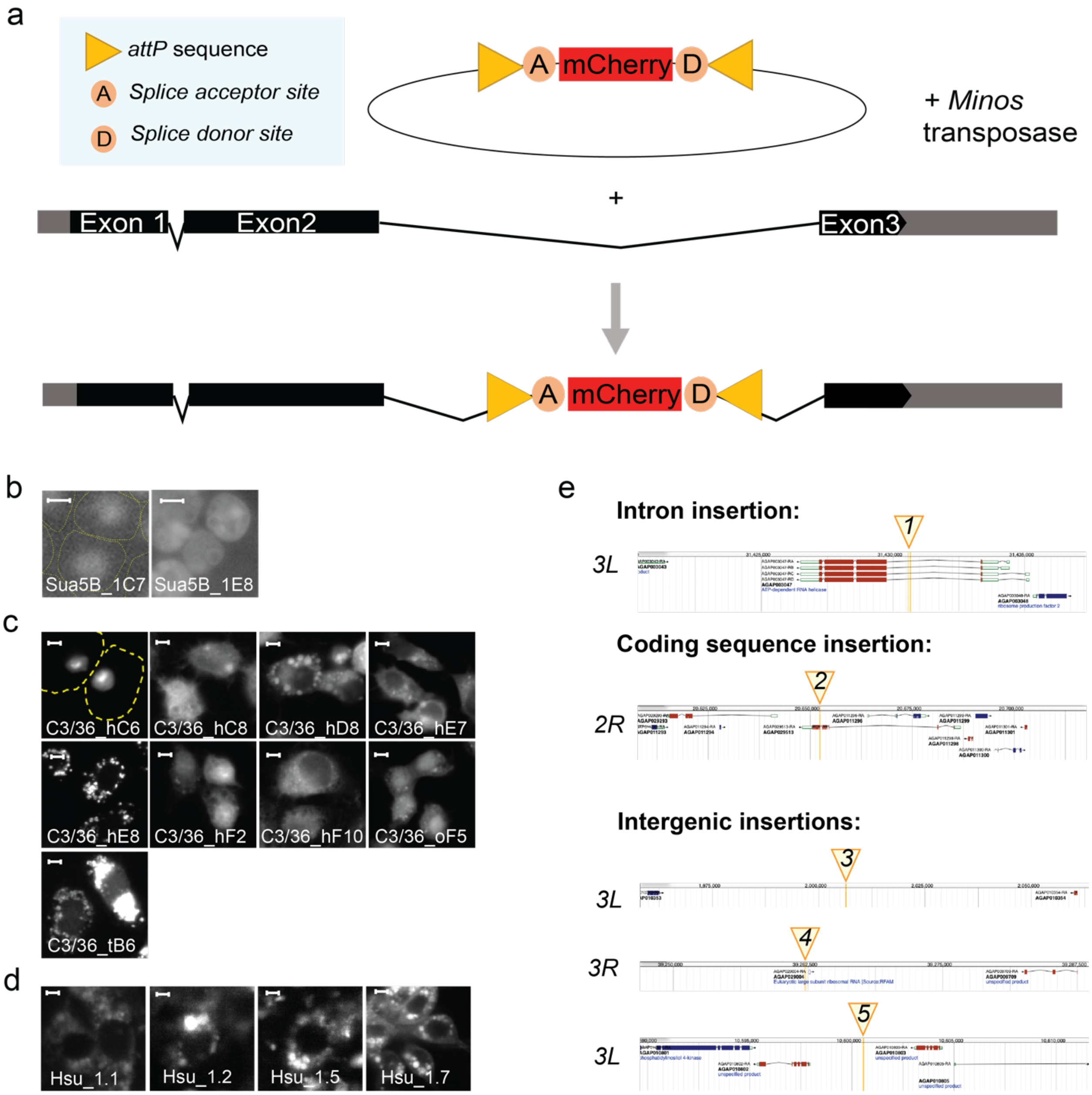

Supplementary Figure 1. **Building mosquito RMCE acceptor cell lines**. (**a**) A *Minos* transposon plasmid with promoterless mCherry was co-transfected along with a *Minos* transposase expression vector to generate intronic insertions that become artificial exons. In rare cases (~0.1%), this successfully results in expression of mCherry as a protein fusion. (**b-d**) mCherry positive *Anopheles* Sua-5B (**b**), *Aedes* C6/36 (**c**), or *Culex* NAMRU2-CQ-01 (Hsu) (**d**) cells were isolated by fluorescence-activated cell sorting (FACS) and iteratively subcloned to obtain isogenic populations with bright mCherry signal. The mCherry-positive cells cloned and imaged by fluorescence microscopy, revealing a variety of intracellular distributions, consistent with exon-tagging of a variety of endogenous genes. (**e**) Mapping of genomic insertions of the MiMIC cassette in the Sua-5B-IE8**-**Act::Cas9-2A-Neo cell line. Reads containing exact match to right or left arm of MiMIC cassette were mapped to AgamP4.12 using BLAST. Scale bar, 5 μm.

SUPPLEMENTARY FIGURE 2

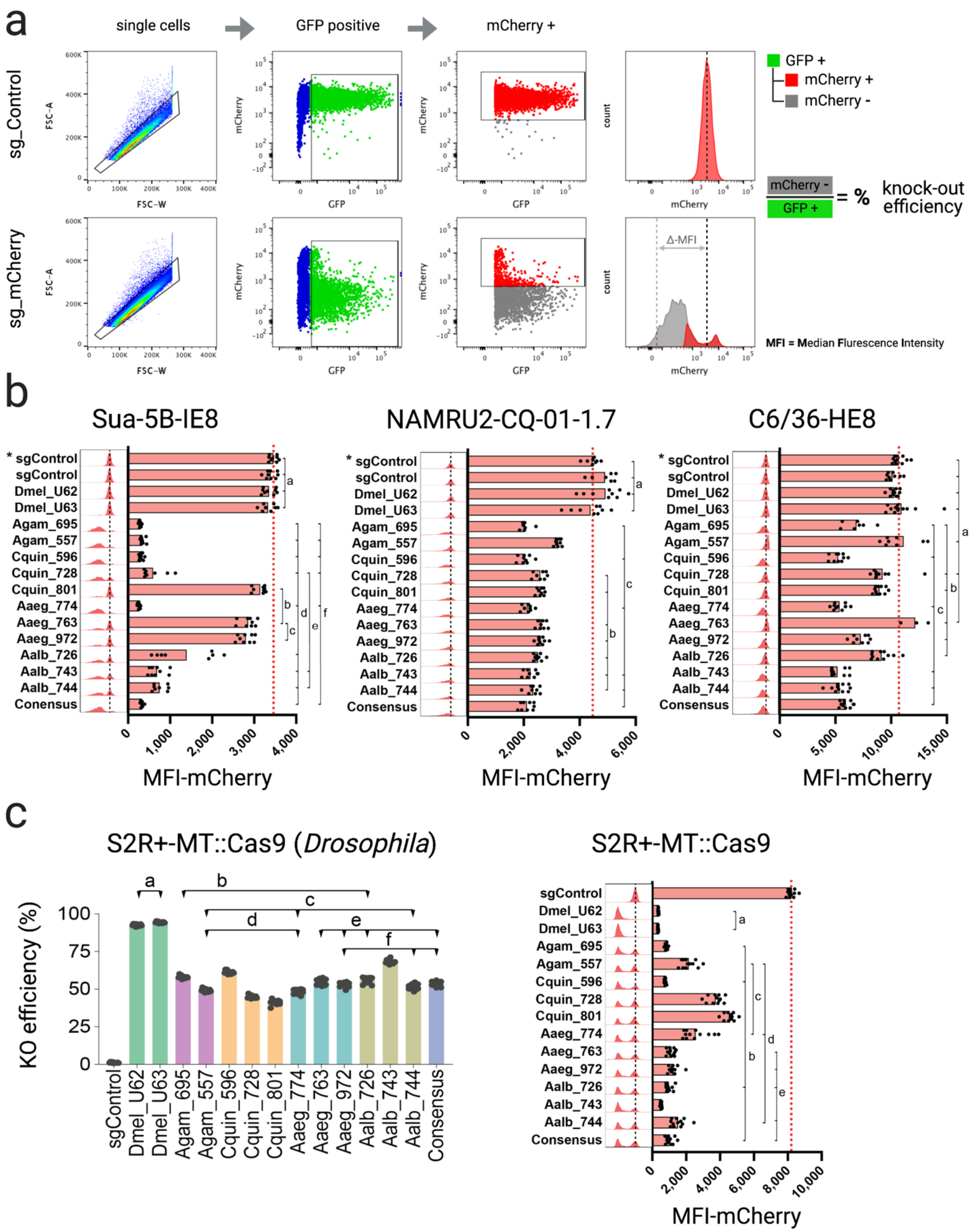

Supplementary Figure 2. Flow cytometry gating strategy and supporting data on KO efficiency with different U6 promoters. (**a**) Schematic detailing the gating strategy used for flow cytometry analysis: single cells were gated based on forward scattering parameters; a subordinate gate was defined

for GFP positive cells based on an un-transfected control; GFP positive population was sub-gated to define mCherry positive cells based on an mCherry-positive/GFP-positive control; GFP positive cells residing out of this gate were categorized as mCherry negative. The % of mCherry negative cells within the GFP population over the total GFP-positive population defines the KO efficiency of the promoter tested. Alternatively, direct quantification of the MFI of the mCherry signal within the GFP-positive population and variation of MFI (ΔMFI) relative to control can be used to infer KO efficiency. The MFI is a parameter of the population correlating directly with the number of mCherry molecules present in each cell, and thus can also be used to quantify KO of the mCherry fluorophore. MFIs are shown as dashed lines in the mCherry population histogram. (**b**) Histograms representing the MFIs of mCherry signals for each U6 promoter tested in the three cell lines. Bars represent the mean value of the MFI and dots represent the distribution of multiple reps obtained from 3 independent experiments. On the y-axis is also plotted a representative example of the mCherry signal fluorescence distribution within the GFP-positive population, with demarcation (dashed line) of the approximate position of the median in control samples. (**c**) Histogram relative to flow cytometry analysis of CRISPR KO efficiency with different U6 promoters in *Drosophila* S2R+-MT::Cas9 cells. On the left: Histogram bars represent the mean, dots represent the distribution of multiple reps obtained from 3 independent experiments. Histogram colors denote the species of origin of the U6 promoters analyzed, shown with abbreviation of species name and three last letters of the corresponding Vectorbase gene ID. On the right: MFIs analysis of mCherry signal. Bars represent the mean value of the MFI and dots represent the distribution of multiple reps obtained from 3 independent experiments. sgControl= pLib6.4-Agam_695 U6 expressing the empty BbsI cassette was used as control. *sgControl refers to the sgControl guide transfected without the Cas9 expressing plasmid. Statistical analysis was performed using Brown-Forsythe and Welch ANOVA tests followed by Dunnett's multiple comparison test. Lowercase letter groupings denote differences not significant ($P_{Dunnett} > 0.05$). All differences between samples of different groupings are significant ($P_{Dunnett} < 0.05$). Raw data, detailed descriptive statistics and statistical analysis, including sample number and calculated P values for each comparison, are reported in Supplementary Data 1 and Source Data 1 files.

SUPPLEMENTARY FIGURE 3

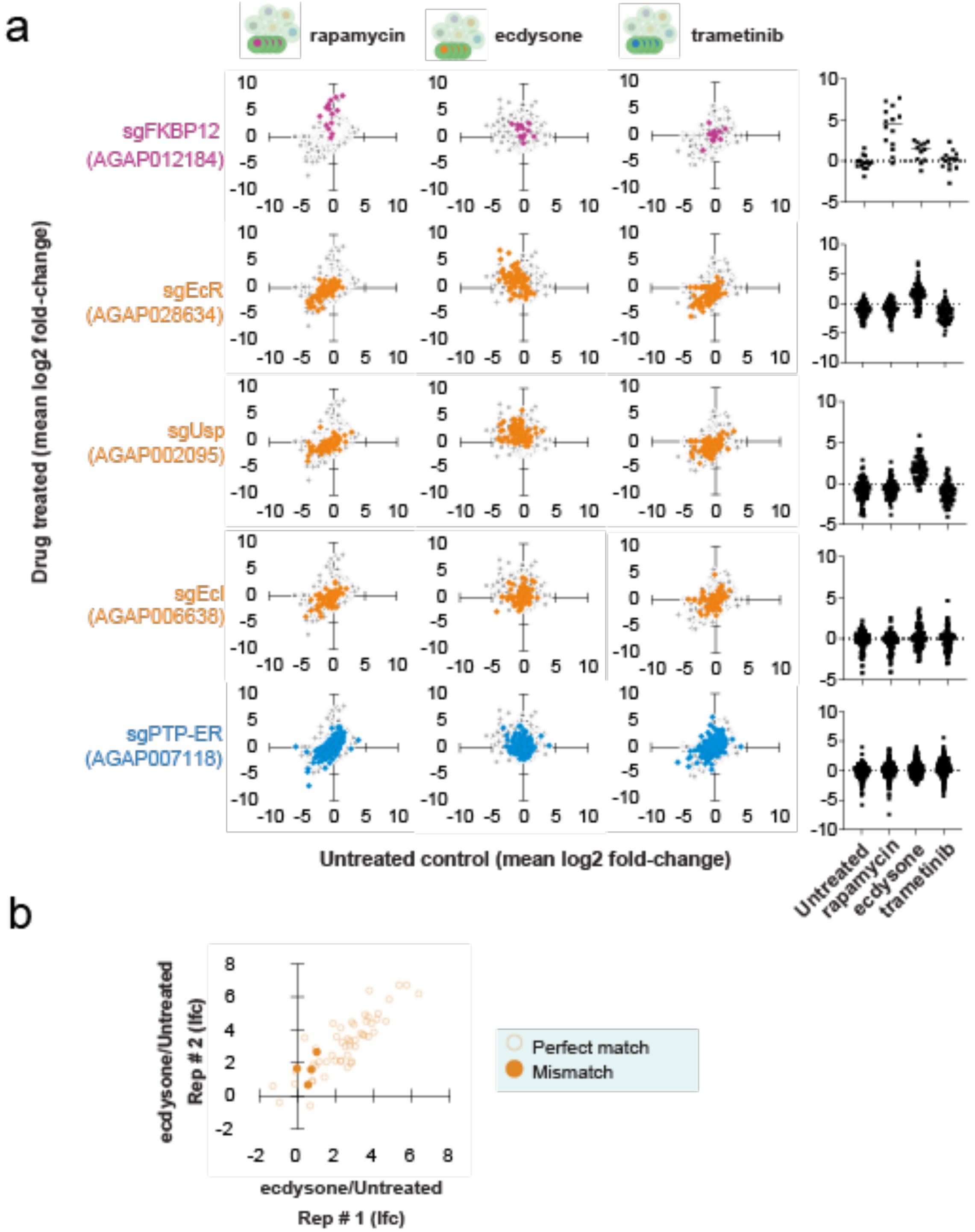

Supplementary Figure 3. **Further analysis of pilot *Anopheles* CRISPR screen data**. (**c**) (Right) Log2 fold-change of sgRNAs targeting indicated gene in drug treated cell population as a function of untreated. (Left) Log2 fold-change of each sgRNA targeting indicated gene in each drug visualized as a staggered bar plot. (**b**) Enrichment of an sgRNA targeting *usp* (AGAP002095) in two biological replicates of the ecdysone screen. Variants in *usp* in Sua5B-IE8-Act::Cas9-2A-Neo cells relative to the reference genome (AgamP4) resulted in mismatches in 4 sgRNAs. Mismatched guides were not enriched following ecdysone treatment.
