# Supplementary material for: Bioinformatic and cell-based tools for pooled CRISPR knockout screening in mosquitos
Source: Nat Commun. 2021 Nov 24;12:6825. doi: 10.1038/s41467-021-27129-3 (PMC8613219; doi:10.1038/s41467-021-27129-3)
Supplement: Supplementary file 2 — Reporting Summary [file 41467_2021_27129_MOESM2_ESM.pdf]

nature portfolio

Corresponding author(s): Norbert Perrimon, Raghuvir Viswanatha
Last updated by author(s): Oct 25, 2021

Reporting Summary

Nature Portfolio wishes to improve the reproducibility of the work that we publish. This form provides structure for consistency and transparency in reporting. For further information on Nature Portfolio policies, see our Editorial Policies and the Editorial Policy Checklist.

Statistics

For all statistical analyses, confirm that the following items are present in the figure legend, table legend, main text, or Methods section.

n/a	Confirmed	
☐	☒	The exact sample size (*n*) for each experimental group/condition, given as a discrete number and unit of measurement
☐	☒	A statement on whether measurements were taken from distinct samples or whether the same sample was measured repeatedly
☐	☒	The statistical test(s) used AND whether they are one- or two-sided<br>*Only common tests should be described solely by name; describe more complex techniques in the Methods section.*
☒	☐	A description of all covariates tested
☐	☒	A description of any assumptions or corrections, such as tests of normality and adjustment for multiple comparisons
☐	☒	A full description of the statistical parameters including central tendency (e.g. means) or other basic estimates (e.g. regression coefficient) AND variation (e.g. standard deviation) or associated estimates of uncertainty (e.g. confidence intervals)
☐	☒	For null hypothesis testing, the test statistic (e.g. *F*, *t*, *r*) with confidence intervals, effect sizes, degrees of freedom and *P* value noted<br>*Give P values as exact values whenever suitable.*
☐	☒	For Bayesian analysis, information on the choice of priors and Markov chain Monte Carlo settings
☐	☒	For hierarchical and complex designs, identification of the appropriate level for tests and full reporting of outcomes
☐	☒	Estimates of effect sizes (e.g. Cohen's *d*, Pearson's *r*), indicating how they were calculated

Our web collection on statistics for biologists contains articles on many of the points above.

Software and code

Policy information about availability of computer code

Data collection	No software was used to collect the data in this study.
Data analysis	All details of data analysis are specified in Methods section. Briefly, open source MAGeCK (versions 0.5.4 or 0.5.6) were used in all CRISPR sequencing analysis. Custom code was used to create CRISPR GuideXpress, which is available at https://www.flyrnai.org/too1s/fly2mosquito/web/. Additional commercial software used was Flow Jo (version 10.7.1, BD; with "DownSample" plugin), Fiji (open source ImageJ2, version 2.1.0/1.53c; alghorithms: "Li"; "Fill holes"; "watershed"), Adobe Illustrator (version 25.2.1), Graph Pad Prism (version 9.1.2), Bio-Rad Image Lab (version 6.1) and Biorender (BioRender 2021).

For manuscripts utilizing custom algorithms or software that are central to the research but not yet described in published literature, software must be made available to editors and reviewers. We strongly encourage code deposition in a community repository (e.g. GitHub). See the Nature Portfolio guidelines for submitting code & software for further information.

Data

Policy information about availability of data

All manuscripts must include a data availability statement. This statement should provide the following information, where applicable:

- Accession codes, unique identifiers, or web links for publicly available datasets
- A description of any restrictions on data availability
- For clinical datasets or third party data, please ensure that the statement adheres to our policy

The raw sequencing reads and VCF files of whole genome sequencing data from Sua-5B-IE8-Act::Cas9-2A-Neo cell line are available for download at CRISPR GuideExpress (https://www.flyrnai.org/too1s/fly2mosquito/web/download) as well as the pilot CRISPR screen result.

Field-specific reporting

Please select the one below that is the best fit for your research. If you are not sure, read the appropriate sections before making your selection.

- [x] Life sciences
- [ ] Behavioural & social sciences
- [ ] Ecological, evolutionary & environmental sciences

For a reference copy of the document with all sections, see nature.com/documents/nr-reporting-summary-flat.pdf

Life sciences study design

All studies must disclose on these points even when the disclosure is negative.

Sample size

For flow cytometry experiments, number of GFP positive gated events analyzed was arbitrarily set to a minimum of 1000, 2000 or 3000 in order to include the highest possible number of samples/technical replicates within the same biological replicative experiment. Considering that in flow cytometry the standard deviation for a gate with 1000 events due to the measurement itself is estimated to be ±3%, this number of events ensures execution of robust statistics (https://doi.org/10.1002/cyto.a.20549). Note that the number of gated events was normalized across all samples within the same biological replicate, and samples including originally more than the threshold set number were "downsized" using a specific "DownSample" plugin available for FlowJo. Downsampling is a common practice in flow cytometry analysis to ensure homogeneous statistic analysis between samples of variable sizes.
For CRISPR screening experiments, >1000 cells per sgRNA were represented at all times, a standard number of cell/sgRNA according to previous studies.

Data exclusions

For flow cytometry analysis, all samples with less than 1000 GFP positive events were discarded from the analysis.

Replication

Flow cytometry experiments were performed in 3 biological replicates with 3 or 4 technical replicates for each sample. For CRISPR screening experiments, two replicate screens were performed for each treatment, and both were reported in the paper. All attempts of replication were successful.

Randomization

This is not relevant to our study because all experimental groups were treated identically and on the same day for each experiment, therefore we expect little batch-to-batch variation and did not require randomization.

Blinding

Our experiments did not include subjective measurements and therefore did not require blinding.

Reporting for specific materials, systems and methods

We require information from authors about some types of materials, experimental systems and methods used in many studies. Here, indicate whether each material, system or method listed is relevant to your study. If you are not sure if a list item applies to your research, read the appropriate section before selecting a response.

Materials & experimental systems

n/a	Involved in the study	
[ ]	[x]	Antibodies
[ ]	[x]	Eukaryotic cell lines
[x]	[ ]	Palaeontology and archaeology
[x]	[ ]	Animals and other organisms
[x]	[ ]	Human research participants
[x]	[ ]	Clinical data
[x]	[ ]	Dual use research of concern

Methods

n/a	Involved in the study	
[x]	[ ]	ChIP-seq
[ ]	[x]	Flow cytometry
[x]	[ ]	MRI-based neuroimaging

Antibodies

Antibodies used

mouse monoclonal anti-mCherry-Tag antibody (St Jonh's Laboratory #STJ34373; clone name not available; this antibody has been discontinued from the manufacturer); rabbit polyclonal anti-flag (Sigma # F7425); goat anti-mouse Alexa Fluor Plus 800 (Thermo Fisher Scientific, #A32730), goat anti-rabbit StarBright Blue 700 (Bio-Rad, #12004161), human Fab anti-actin rhodamine-conjugated (Bio-Rad, #12004164).

Validation

Human Fab anti-actin rhodamine-conjugated (Bio-Rad, #12004164) was validated by the manufacturer to be effective against human, mouse and rat beta-actin. We did extensively validated through western blot cross-reactivity of this antibody with Drosophila melanogaster, Anopheles coluzzii, Aedes albopictus and Culex quinquefasciatus beta-actin. In all species tested the blot revealed a unique clean signal corresponding to actin MW.
Mouse monoclonal anti-mCherry-Tag antibody (St Jonh's Laboratory #STJ34373-now discontinued), was validated for use in western blotting by the manufacturer, and tested in this work with Anopheles cells, yielding no background and a clean signal only in mCherry expressing cell lines.

Eukaryotic cell lines

Policy information about cell lines

Cell line source(s)	Sua-5b cells (Anopheles coluzzi/gambiae; RRID:CVCL_RQ24) were provided by F. Catteruccia (Harvard T.H. Chan School of Public Health). NAMRU2-CQ-01 cells (Culex quinquefasciatus; RRID:CVCL_1B68) were provided by Nelson Lau (Boston University). C6/36 cells (Aedes albopictus; RRID:CVCL_Z230) were provided by Tonya Colpitts (Boston University). S2R+-MT::Cas9 cells (Drosophila melanogaster; RRID:CVCL_UD30) were available in the Perrimon lab.
Authentication	For Sua-5b cells diagnostic PCR was performed following the protocol specified in Santolamazza et al.10 for Anopheles M/S molecular form discrimination confirming the presence of the SINE200 insertion specific to the M form (currently recognized as the Anopheles coluzzii species). Variant calling analysis was performed by comparing whole-genome sequence data from the Sua-5b-IE8-Act::Cas9-2A-Neo cell line to Anopheles gambiae (AgamP4) or Anopheles coluzzii (AcolM1.8) genome sequence. See methods for more information.
Mycoplasma contamination	Cells were mycoplasma-free as confirmed by diagnostic PCR following established protocols.
Commonly misidentified lines (See ICLAC register)	No commonly misidentified cell liones were used in this study.

Flow Cytometry

Plots

Confirm that:

- [x] The axis labels state the marker and fluorochrome used (e.g. CD4-FITC).
- [x] The axis scales are clearly visible. Include numbers along axes only for bottom left plot of group (a 'group' is an analysis of identical markers).
- [x] All plots are contour plots with outliers or pseudocolor plots.
- [x] A numerical value for number of cells or percentage (with statistics) is provided.

Methodology

Sample preparation	Following transfection and outgrowth, live cells in growth media (Schneider's media containing 10% FBS + P/S) were subjected to flow cytometry analysis of live-cell fluorescent reporters.
Instrument	FACSymphony analyzer (BD)
Software	BD FACS Diva and Flow Jo (version 10.7.1)
Cell population abundance	Fraction of gated population (i.e., transfected cells) varied depending on transfection efficiency, and ranged from 1-20% of starting population.
Gating strategy	Gates for cell singlets were defined based on forward and side scattering beams and a subordinate gate for GFP+ cells was defined based on untransfected control cells. GFP+ cells were sub-gated to define mCherry+ cells and mCherry- cells within the GFP population were defined by exclusion from the mCherry+ gate (NOT Boolean gate for mCherry+).

- [x] Tick this box to confirm that a figure exemplifying the gating strategy is provided in the Supplementary Information.
