# Supplementary material for: Bioinformatic and cell-based tools for pooled CRISPR knockout screening in mosquitos
Source: Nat Commun. 2021 Nov 24;12:6825. doi: 10.1038/s41467-021-27129-3 (PMC8613219; doi:10.1038/s41467-021-27129-3)
Supplement: Supplementary file 3 — Description of Additional Supplementary Files [file 41467_2021_27129_MOESM3_ESM.pdf]

Title: Supplementary Data 1- List of primers, vectors and cell lines

Description: Includes a list of primers referenced in the Methods; all plasmids built or used for this study; parental and new engineered cell lines; sequences and features of U6 promoter expressing cassette used to build the attB library donor vectors. The data as described is subdivided in the following tabs:

1. **Primers**
2. **Plasmids**
3. **Cell lines**
4. **Selected U6 promoter sequences:** Includes the sequence incorporated in the attB-library donor vectors and other structural details.

Title: Supplementary Data 2- Flow cytometry analysis data

Description: Data relevant to U6 promoter evaluation (data represented in **Fig.3c,d,e** and **Supplementary Figure 2b,c**). Includes output from FlowJo analysis and calculation of KO efficiency relative to the three mosquito cell lines analyzed. The datasets for each cell line is subdivided in a separate tabs:

1. **Sua-5B-IE8**
2. **NAMRU2-CQ-01-1.7**
3. **C6/36-HE8**
4. **S2R+-MT::Cas9**

Title: Supplementary Data 3- Anopheles sgRNA Library and Readcounts

Description: Data relevant to *Anopheles* CRISPR screen (data represented in **Figures 5c-e** and **Supplementary Figure 3)**. Includes sgRNA library, readcount, analysis, and primers. The data as described is subdivided in the following tabs:

1. **sgRNA library**
2. **CRISPRseq Primers**
3. **Readcounts**
4. **Log2 fold-change**
5. **MaGeCK Test**
6. **Variant analysis**

Title: Supplementary Data 4- CRISPR GuideXpress sgRNA design statistics

Description: Includes raw statistics regarding sgRNA designs for all the supported species and data represented in **Figure 1b-e**. The data as described is contained in a single tab:

1. **Raw statistics**
